# Lifetime Management of Transcatheter Aortic Valve Replacement: A Guide to Decision-Making and Future Reinterventions

**DOI:** 10.3390/jcm15051917

**Published:** 2026-03-03

**Authors:** Malanka Lankaputhra, Dion Stub, Riley J. Batchelor, Vishal Goel, Nay Min Htun

**Affiliations:** 1 School of Public Health and Preventive Medicine, Monash University, Melbourne, VIC 3004, Australia; 2 Department of Cardiology, The Alfred Hospital, Melbourne, VIC 3004, Australia; 3 Baker Heart and Diabetes Institute, Melbourne, VIC 3004, Australia; 4 Department of Cardiology, Victorian Heart Hospital, Clayton, VIC 3168, Australia; 5 Department of Cardiology, Peninsula Health, Langwarrin, VIC 3910, Australia

**Keywords:** transcatheter aortic valve replacement, lifetime management, computed tomography, redo-TAVR, coronary obstruction

## Abstract

Transcatheter aortic valve replacement (TAVR) has revolutionized the treatment of severe aortic stenosis, evolving from a therapy reserved for inoperable patients to a first-line option across all surgical risk categories. As TAVR expands to younger patients with longer life expectancies, lifetime management strategies become paramount. This comprehensive review examines the important role of computed tomography (CT) planning in optimizing initial valve selection and predicting future reintervention feasibility. We discuss the decision framework between TAVR and surgical aortic valve replacement (SAVR) as initial therapy, strategies to optimize the index TAVR procedure, including minimizing patient-prosthesis mismatch, reducing paravalvular regurgitation, preventing conduction abnormalities and coronary obstruction, and facilitating future reinterventions. For patients requiring redo procedures, we analyse TAVR-in-TAVR considerations, including risk plane assessment, coronary access preservation, and leaflet modification techniques. Future directions include advances in valve design, artificial intelligence integration in procedural planning, and development of personalized risk assessment tools. Successful lifetime management requires multidisciplinary collaboration and individualized treatment planning to optimize outcomes throughout a patient’s lifetime journey with aortic valve disease.

## 1. Introduction

Severe aortic stenosis represents the most prevalent valvular disease in developed nations, with an estimated prevalence of 2–7% in patients over 65 years of age [1]. The management landscape has undergone a dramatic transformation since the first transcatheter aortic valve replacement was performed in 2002. Initially reserved for inoperable or high-risk surgical candidates, TAVR has demonstrated non-inferiority or superiority to surgical aortic valve replacement across all risk strata, fundamentally altering treatment paradigms [2,3,4,5].

The expansion of TAVR to younger, low-risk patients introduces complex considerations regarding valve durability and the potential need for multiple interventions over a patient’s lifetime. Current guidelines reflect this evolution, with the American College of Cardiology/American Heart Association recommending TAVR for patients aged >80 years and suggesting shared decision-making for those aged 65–80 years, while European Society of Cardiology/European Association for Cardio-Thoracic Surgery guidelines suggest TAVR in patients aged ≥70 years and SAVR in younger patients at low surgical risk [3,6,7]. As patients increasingly outlive their first transcatheter heart valve (THV), the concept of lifetime management emerges as a vital framework for optimizing long-term outcomes.

This comprehensive review examines the multifaceted aspects of lifetime management in TAVR, emphasizing the pivotal role of preprocedural planning, the decision framework between TAVR and SAVR, optimization strategies for the index procedure, and management of future reinterventions.

## 2. Materials and Methods

This narrative review addresses lifetime management considerations for patients undergoing aortic valve replacement in an era of expanding TAVR indications. The objective was to provide a practical, imaging-centered framework to support initial strategy selection (TAVR versus SAVR), index procedural planning, and anticipation of future reintervention feasibility.

A targeted literature search was conducted in MEDLINE/PubMed, Embase, and the Cochrane Library (January 2010 to January 2026), supplemented by hand-searching reference lists of contemporary guidelines, consensus statements, and landmark trials. Article selection prioritized clinical practice guidelines, randomised controlled trials, large registries, and well-characterized observational cohorts. Bench-testing and computational modelling studies were included where they directly informed geometric feasibility assessments and are explicitly identified as such. Case reports and first-in-human series were included selectively for emerging techniques, with their preliminary evidence base acknowledged. No formal quality appraisal, meta-analysis, or quantitative synthesis was performed.

## 3. Decision Framework: TAVR vs. SAVR as Initial Therapy

The choice between TAVR and SAVR as initial therapy represents a decision point that influences all subsequent interventions. Current guidelines provide some framework recommendations, largely based on age, while acknowledging the complexity of individual decision-making [3,6]. However, age alone provides insufficient guidance, as physiological age, comorbidities, and life expectancy more accurately inform decision-making [8,9].

The TAVR-first approach offers immediate benefits, including shorter hospitalization, faster recovery, and avoidance of sternotomy [10]. These advantages particularly appeal to younger, active patients seeking rapid return to normal activities. Recent data from low-risk trials demonstrate excellent short-term outcomes, with the PARTNER 3 trial showing lower rates of death, stroke, or rehospitalization at 1 year with TAVR compared to SAVR (8.5% vs. 15.1%, *p* < 0.001) [2]. Although the transcatheter valve carries a higher upfront device cost, shorter intensive care and hospital stays may partially offset this difference. These potential savings must be weighed against uncertainties in long-term durability, and the added complexity and cost of future reinterventions should structural valve deterioration occur.

The SAVR-first strategy provides established long-term durability data and preserves straightforward options for future valve-in-valve TAVR when the surgical bioprosthesis degenerates [10]. Modern surgical techniques, including minimally invasive approaches and rapid deployment valves, have reduced perioperative morbidity [11]. Moreover, SAVR in young patients offers the advantage of performing surgery when surgical risk is lowest, with estimated in-hospital mortality of 1.4% among low-risk patients [11].

**Figure 1:**
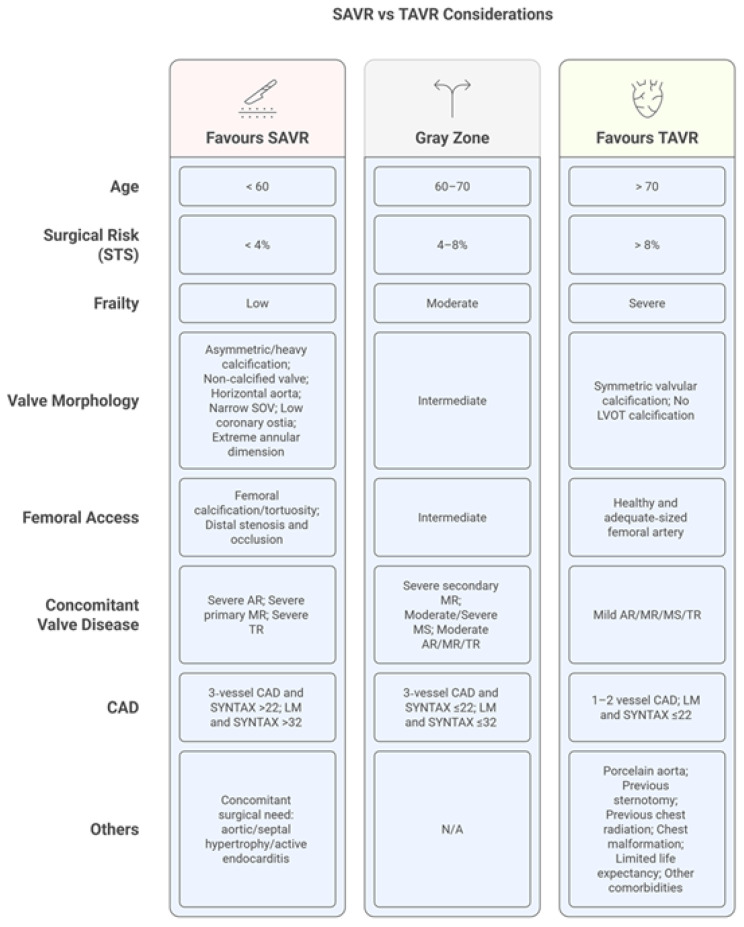
Factors affecting decision making regarding TAVR and SAVR.

**Figure 2:**
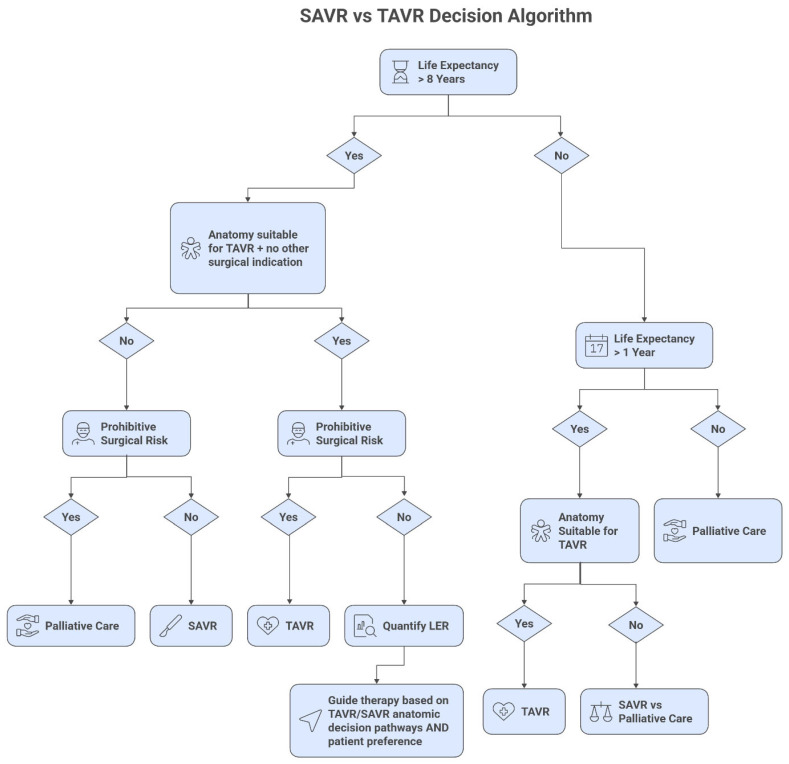
Decision-making framework for selecting between transcatheter and surgical aortic valve replacement strategies. This algorithm integrates patient-specific factors with lifetime management considerations to optimize initial intervention selection. Key decision points include surgical risk assessment, concurrent coronary disease requiring bypass grafting, and projected lifetime reintervention probability. Abbreviations: TAVR (transcatheter aortic valve replacement), SAVR (surgical aortic valve replacement), CABG (coronary artery bypass grafting), LER (lifetime estimated risk). Adapted from Chen et al. with permission [12].

Several clinical and anatomical factors influence this decision. Clinical factors favoring TAVR include higher surgical risk, previous cardiac surgery, frailty, and patient preference for less invasive therapy. Anatomical features favoring TAVR include porcelain aorta, chest deformity, and patent coronary grafts at risk during redo sternotomy [10]. Conversely, SAVR should be preferred in patients with bicuspid aortic valves (particularly Sievers type 0), extensive coronary disease requiring bypass surgery, other valvular disorders requiring intervention, or ascending aortic pathology (Figure 1 and Figure 2) [10,12,13].

Importantly, the initial intervention has major implications for subsequent procedures. As Jubran et al. note, approximately 20% of patients require reintervention within their lifetime, making the feasibility of future procedures a key consideration [14,15]. The concept of lifetime estimated risk, incorporating cumulative procedural risks over a patient’s expected lifespan, should guide decision-making rather than focusing solely on immediate procedural risk [11].

## 4. Optimizing the Index TAVR Procedure

When TAVR is selected as the initial strategy, meticulous preprocedural planning and execution are essential to maximize durability and preserve future reintervention options, with CT serving as the cornerstone imaging modality for most of these considerations [16].

Throughout this review, commonly referenced CT-derived measurements and thresholds are presented as pragmatic planning guides rather than universal cut-offs. Their predictive value varies with valve platform design (including frame height, sealing skirt configuration, and commissural architecture), implantation technique (including depth and commissural alignment), and patient-level anatomy. They should therefore be interpreted within the broader anatomical and clinical context rather than in isolation. A summary of recommendations is provided in Table 1.

## 5. Patient-Prosthesis Mismatch

Patient-prosthesis mismatch represents a critical complication where the prosthetic valve’s effective orifice area proves inadequate relative to the patient’s body surface area. The condition is stratified by indexed effective orifice area (iEOA), with values below 0.85 cm^2^/m^2^ indicating PPM and those under 0.65 cm^2^/m^2^ defining severe mismatch [15]. Modern transcatheter valve technology and enhanced CT-guided sizing protocols have substantially reduced severe PPM incidence to approximately 6.3% with contemporary devices [16].

Patients presenting the greatest PPM risk include those with extreme body habitus or diminutive aortic anatomy. Key anatomical predictors identified on CT imaging encompass annular areas under 400 mm^2^, perimeter measurements below 70 mm, and mean diameters less than 20 mm. Individuals with body surface areas exceeding 2.0 m^2^ combined with annular dimensions under 430 mm^2^ demonstrate elevated risk profiles, particularly when extensive calcification further compromises the functional orifice area [16]. These thresholds are drawn from observational cohorts and should be interpreted in the context of valve platform and predicted hemodynamics.

The landmark publication by Hahn and colleagues established definitive hemodynamic reference values for various transcatheter valve designs and sizes, now serving as the foundation for PPM risk assessment [17]. Balloon-expandable SAPIEN 3 prostheses demonstrate predicted effective orifice areas spanning 1.1–1.2 cm^2^ for the smallest 20 mm device up to 2.0–2.2 cm^2^ for the largest 29 mm valve. Self-expanding platforms achieve superior hemodynamic profiles through their supra-annular leaflet positioning, with Evolut valves typically providing 15–20% greater effective orifice areas compared to equivalent balloon-expandable sizes, offering particular advantages in challenging small annuli or larger patients [17].

Optimal CT-based measurement techniques require acquisition during systole (specifically 20–30% of the cardiac cycle) to capture maximum annular dimensions [18]. Planning algorithms should incorporate area-derived sizing for balloon-expandable platforms and perimeter-based calculations for self-expanding devices. When encountering small annuli (defined as areas under 400 mm^2^ or perimeters below 70 mm), supra-annular self-expanding prostheses emerge as the preferred option. Risk stratification employs the Hahn equation (predicted EOA divided by BSA) [17], and when calculations suggest severe PPM likelihood despite optimal transcatheter options, surgical intervention with annular enlargement warrants consideration.

Limitations of CT-based sizing should be recognized. Image quality can be reduced by motion (e.g., atrial fibrillation), heavy calcification, and blooming artefact from pre-existing metallic implants (such as coronary stents, surgical rings, or pacemaker/ICD leads). When annular or LVOT delineation is compromised, optimization strategies include ECG-gating, iterative reconstruction/metal artefact reduction, dual-energy acquisition where available, and confirmation with complementary imaging (e.g., 3D transesophageal echocardiography).

Recent evidence from the SMART trial reinforces the hemodynamic advantages of self-expanding technology in constrained anatomy, demonstrating reduced PPM and improved valve function in annuli measuring 430 mm^2^ or smaller [19]. Patient body habitus significantly influences device selection: individuals with BSA exceeding 2.0 m^2^ should generally receive self-expanding valves when annular dimensions fall below 450 mm^2^, while those above 2.2 m^2^ BSA benefit from self-expanding technology regardless of anatomy. Patients presenting with BSA greater than 2.5 m^2^ and annular areas under 500 mm^2^ may require surgical intervention with root modification as the sole strategy to prevent severe mismatch [16,19].

## 6. Minimizing Paravalvular Regurgitation

Contemporary valve designs have substantially reduced paravalvular leak through improved sealing mechanisms and refined sizing protocols, yet comprehensive CT evaluation of calcification burden, distribution, and annular morphology remains central to predicting and preventing this complication [16].

Calcium burden is the primary determinant of paravalvular regurgitation (PVL). Total aortic valve complex calcium volumes exceeding 500 mm^3^ are associated with increased PVL risk, particularly when asymmetrically distributed, such as when more than 50% of the total burden is concentrated within a single sector [20,21]. LVOT calcification extending more than 5 mm below the annular plane and very dense deposits (greater than 850 HU) further compound this risk [20,21]. These thresholds should be interpreted in the context of the chosen platform’s radial force and sealing skirt design.

The spatial distribution of calcium carries independent predictive significance. Commissural calcium exceeding 3 mm in thickness compromises inter-commissural sealing, near-circumferential annular involvement (greater than 75%) is associated with increased PVL, and bulky nodular deposits protruding more than 4 mm into the outflow tract may prevent full frame apposition [20]. A volume differential exceeding 300 mm^3^ between opposing sectors can produce eccentric frame expansion and residual regurgitant jets [20].

Annular eccentricity, quantified by the ellipticity index (maximum minus minimum diameter, divided by maximum diameter), informs platform selection [16]. Values above 0.25 may favor self-expanding devices, given their greater conformability, while values above 0.30 are associated with higher PVL rates with balloon-expandable prostheses [16].

Sizing should be individualized according to calcium burden. For minimal calcification (less than 250 mm^3^), oversizing of 5 to 10% (balloon-expandable) and 15 to 20% (self-expanding) is generally well tolerated. For intermediate burden (250 to 500 mm^3^), balloon-expandable oversizing is typically limited to 10% or less. For extensive calcification (greater than 500 mm^3^), minimal balloon-expandable oversizing (0 to 5%) is preferred to reduce rupture risk while preserving adequate radial force [16,21]. The cover index (100 multiplied by prosthesis minus mean annular diameter, divided by prosthesis diameter) serves as a complementary sizing metric, with target ranges of 5 to 15% for balloon-expandable and 15 to 25% for self-expanding platforms [22]. Values below 5% predict inadequate sealing, whereas balloon-expandable indices above 20% are associated with increased annular disruption risk [22,23].

Several anatomical variants necessitate modified planning. Bicuspid morphology is associated with higher PVL rates and often requires multi-level annular measurements; balloon-expandable platforms may be preferred in selected cases for their more predictable circular expansion [13]. An LVOT tapering ratio below 0.85 may enhance prosthesis sealing, whereas ratios above 0.95 can compromise apposition. Aortic angulation exceeding 48 degrees increases the risk of deployment malalignment [13].

In high-risk anatomy, CT-guided procedural modifications can further mitigate PVL. Very high calcium volumes (greater than 750 mm^3^) may warrant pre-dilatation at approximately 90% of the mean annular diameter to facilitate uniform expansion [15,20]. Extensive LVOT calcification may favor a higher deployment position (80% aortic, 20% ventricular) to minimize frame interaction with subannular calcium. A low cover index (below 8%) should prompt anticipatory planning for targeted post-dilatation, using a balloon not exceeding the nominal prosthesis size and directed at the jet location identified on preprocedural imaging [16,20].

## 7. Preventing Conduction Abnormalities

The cardiac conduction system is inherently vulnerable during TAVR owing to the anatomical proximity of the bundle of His along the inferior margin of the membranous septum, where it is susceptible to compression by the expanding valve frame. Permanent pacemaker implantation occurs in 5 to 25% of cases, depending on valve platform and implantation technique, and carries prognostic implications through ventricular dyssynchrony and lead-related tricuspid regurgitation [16]. Minimizing conduction injury is therefore of particular importance in younger patients for whom the cumulative burden of pacing dependency over a longer life expectancy may be substantial.

Contemporary CT imaging enables precise visualization of the membranous septum within the interleaflet triangle between non-coronary and right coronary cusps. The infra-annular membranous septum length (IA-MSL) serves as a validated predictor of conduction disturbances. Rao and colleagues’ standardized measurement technique positions the crosshair between cusps on systolic axial images, isolating the interleaflet triangle for accurate IA-MSL measurement on stretched-vessel reconstructions [15].

Clinical predictors of pacemaker requirement include a short membranous septum (commonly less than 3 mm in observational series), implantation depth exceeding the membranous septum length, and LVOT calcium concentrated at the non-coronary to right-coronary cusp interface [16,21]. These relationships are both platform- and technique-dependent: pacemaker risk increases with deeper implantation, greater radial force, and post-dilatation, and has been attenuated by higher, more controlled deployment strategies such as the cusp-overlap technique for self-expanding valves [16,24]. A substantial proportion of early conduction disturbances resolve during follow-up, reinforcing the importance of structured rhythm surveillance and individualized pacing decisions rather than reflexive permanent pacemaker implantation.

When permanent pacing is required after TAVR, leadless pacemakers represent an increasingly adopted alternative, particularly in older or frail patients in whom avoidance of a surgical pocket and transvenous lead-related complications is desirable. In a Medicare analysis comparing leadless with transvenous systems implanted after TAVR, leadless pacemakers were associated with lower in-hospital complication rates and fewer mid-term device-related complications, with comparable mortality [25]. Broader leadless registries, including i-LEAPER, corroborate these safety findings across patient subgroups [26]. Patient selection remains important, as current leadless platforms provide single-chamber ventricular pacing only and are not suitable when atrial pacing, cardiac resynchronization therapy, or defibrillator capability is required.

Anatomical variants significantly influence risk profiles. The protective variant (30% prevalence) features the His bundle coursing deep within muscular septum, shielding it from compression. Conversely, the “naked” His variant (20% prevalence) positions the bundle superficially beneath minimal endocardial coverage, creating extreme vulnerability even with conservative implantation [15,19].

Novel computational modelling enhances risk prediction by simulating THV forces on the LVOT [15]. Maximum contact pressure and the contact pressure index (proportional area subjected to force) demonstrate strong correlation with pacemaker likelihood, enabling personalized procedural planning. Integration of IA-MSL measurements, anatomical variant recognition, and computational simulation into routine CT protocols allows operators to modify deployment strategies, accepting slightly higher positions in short membranous septa or selecting balloon-expandable valves for naked His variants, ultimately preserving conduction integrity while maintaining procedural success [15,19].

## 8. Preventing Coronary Obstruction

Coronary obstruction represents an infrequent yet life-threatening TAVR complication with an incidence of 0.6% in native valve procedures and mortality rates approaching 50%. The mechanism involves native leaflet displacement toward coronary ostia or, less frequently, direct occlusion by the prosthetic frame. Obstruction patterns include direct leaflet-mediated occlusion and indirect sinus sequestration through sinotubular junction sealing [20,27].

Accurate risk assessment requires systematic CT evaluation of the complex anatomical relationships between coronary origins, sinus dimensions, leaflet characteristics, and sinotubular junction anatomy. Several risk models derived from observational cohorts and CT-based virtual valve simulation incorporate parameters such as leaflet length relative to coronary height, virtual valve-to-coronary (VTC) distance, and leaflet calcium burden [11,28]. A VTC < 4 mm is widely used as a practical marker of high risk, particularly for valve-in-valve procedures, but the optimal threshold is influenced by valve frame design, sealing behaviour, implantation height, and commissural alignment.

Key anatomical predictors encompass [10,21,22]:

- Coronary origins below 12 mm from the annular plane
- Sinotubular junction or sinus width under 30 mm
- Extensive leaflet calcification with bulky morphology
- Diminutive sinus of Valsalva volume
- Reduced sinotubular junction height

Additional procedural factors amplify risk, including elevated deployment positions and valve-in-valve procedures within surgical bioprostheses. Supra-annular transcatheter designs with taller commissural architecture demonstrate higher obstruction potential compared to intra-annular platforms. Although commissural misalignment in self-expanding devices (particularly Evolut and ACURATE neo2) maximizes risk, contemporary alignment techniques partially mitigate this concern [15].

High-risk anatomy necessitates adjunctive protective strategies. Chimney stenting preserves coronary perfusion by extending a stent from the coronary ostium into the ascending aorta, while BASILICA (Bioprosthetic or Native Aortic Scallop Intentional Laceration to Prevent Iatrogenic Coronary Artery Obstruction) [29,30] employs electrocautery-mediated leaflet laceration to create a channel for coronary flow. Early outcomes suggest comparable acute success between techniques, although longer-term comparative data remain limited [16,31,32,33]. Additional leaflet modification approaches under investigation include UNICORN (Undermining Iatrogenic Coronary Obstruction with Radiofrequency Needle) [34] and the dedicated ShortCut device [35]. The evidence base for these newer techniques remains confined to first-in-human experience and early observational series, and reported procedural success rates should not yet be considered broadly generalizable.

## 9. Preventing Annular Rupture

Aortic annular disruption remains an uncommon but catastrophic complication occurring predominantly with balloon-expandable platforms. The dynamic nature of aortic anatomy produces cyclical dimensional changes, with systolic measurements providing maximum values for sizing calculations. Platform-specific algorithms differ substantially: balloon-expandable devices utilize area-based measurements with conservative 0–10% oversizing, while self-expanding valves employ perimeter calculations permitting 10–25% oversizing [10,20].

Left ventricular outflow tract calcification represents the primary risk determinant, particularly nodular deposits near the left fibrous trigone and commissural regions. Balloon-expandable valves demonstrate highest rupture rates in this setting, whereas self-expanding platforms rarely cause disruption except with aggressive balloon manipulation [15,23].

Balloon utilization requires adherence to specific parameters [10,15,22,24]:

- Maximum diameter should not surpass the smaller of mean LVOT or STJ dimensions
- Semicompliant balloons permit 1:1 vessel ratios
- Non-compliant devices require ratios below 1.0

Extensive outflow tract calcium, especially nodular deposits in vulnerable locations, favors self-expanding valve selection. When balloon-expandable devices remain necessary, conservative oversizing (maximum 5%) with meticulous attention to adjunctive balloon dimensions becomes mandatory [15,35,36].

## 10. Optimizing Future TAVR-in-TAVR Feasibility

Long-term management requires consideration of reintervention options at the time of the index procedure. The “risk plane” (neoskirt height) is a useful geometric construct describing the cylindrical zone created when a second valve pins the index leaflets against the aortic wall. It can be estimated as leaflet (or commissural) height minus implantation depth and provides a first-pass assessment of redo-TAVR feasibility and coronary accessibility [16].

In real-world planning, procedural feasibility is determined by more than the commissural post–coronary relationship alone. Operators also consider (i) valve-to-sinotubular junction (VTSTJ) clearance as a marker of sinus sequestration risk, (ii) the virtual valve-to-coronary (VTC) distance after simulated redo deployment, and (iii) whether the target implantation depth of the index valve is realistically achievable for the chosen platform while balancing haemodynamics and conduction safety. Commissural alignment at the index procedure can improve the likelihood of future coronary re-access, particularly with self-expanding valves.

Platform-specific leaflet configurations determine risk plane calculations:

- SAPIEN 3 balloon-expandable: leaflets terminate at commissural tab apex
- Self-expanding designs (Evolut, ACURATE neo2, Portico): leaflets reach commissural post summit
- Supra-annular architectures generate substantially taller risk planes

Optimal strategies aim to keep the neoskirt/risk plane below the coronary ostia or, at minimum, below the STJ. When the projected risk plane lies above the coronaries but below the STJ, redo feasibility depends on adequate valve-to-coronary (VTC) distance and the ability to cannulate through frame cells; pragmatic thresholds such as VTC > 4 mm are commonly used, but are valve- and technique-dependent [16,36]. When the risk plane extends above the STJ, both coronary clearance and valve-to-STJ clearance become critical (e.g., VTC > 4 mm and VTSTJ > 2 mm in some virtual simulation datasets) [16,36].

**Figure 3:**
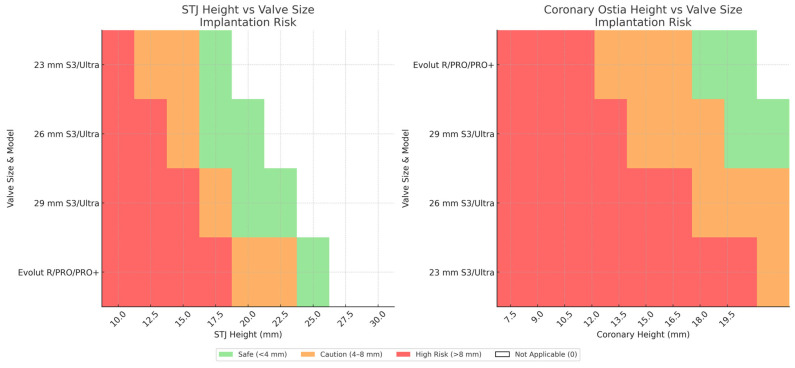
Optimal implantation depth recommendations for maintaining the risk plane below critical anatomical structures during index TAVR procedures. The risk plane represents the commissural post height minus deployment depth. Colour coding indicates conduction risk stratification: green denotes safe deployment (<4 mm) with minimal conduction risk; orange suggests moderate risk (4–8 mm) requiring careful monitoring; red indicates high conduction risk or technically unfeasible depths (>8 mm). For moderate and high-risk deployments, procedural feasibility requires valve-to-sinotubular junction clearance > 2 mm and valve-to-coronary distance > 4 mm to preserve future redo-TAVR options. Device abbreviations: S3 (SAPIEN 3), STJ (sinotubular junction), TAVI (transcatheter aortic valve implantation), VTC (valve-to-coronary), VTSTJ (valve-to-STJ), Ultra (SAPIEN Ultra).

**Figure 4:**
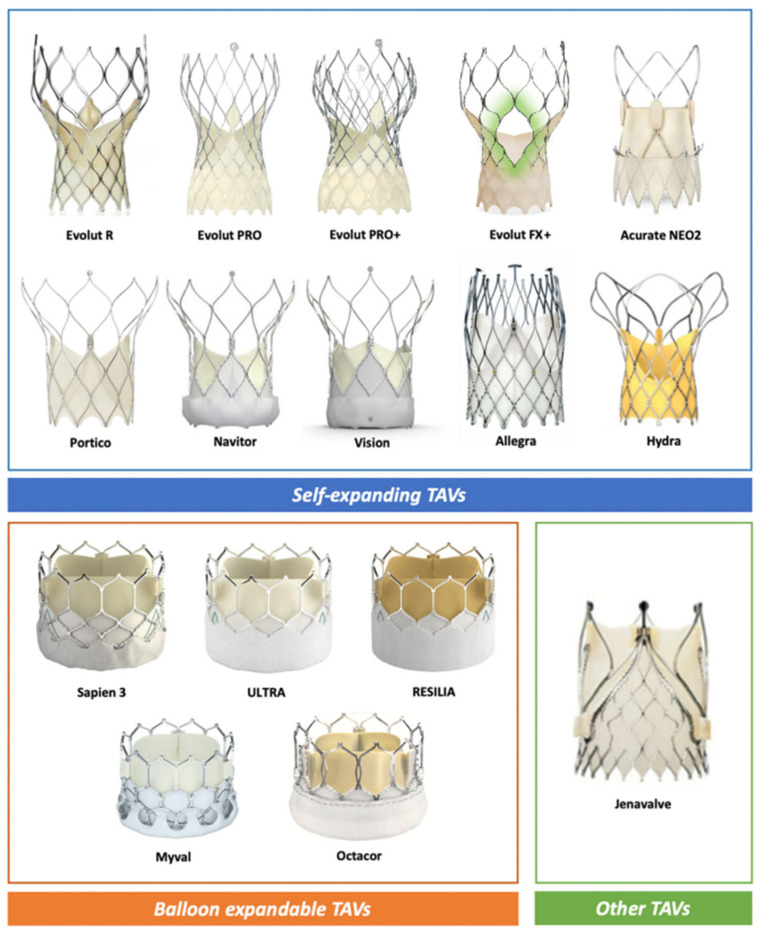
Currently available transcatheter aortic valve platforms. Adapted from Valvo et al. [37] with permission.

Figure 3 provides implantation depth recommendations for optimizing risk plane positioning relative to critical anatomical landmarks. These evidence-based parameters enable operators to balance immediate procedural success with preservation of future treatment options, exemplifying the evolution toward lifetime management strategies in structural heart disease. Figure 4 describes most currently available valve platforms.

## 11. Optimizing the Index SAVR Procedure

The selection of aortic valve replacement strategy represents one of the most complex decisions in cardiac surgery, typically requiring multidisciplinary heart team evaluation to balance immediate outcomes with lifetime management considerations [31]. For younger patients, mechanical valve replacement offers durability but mandates lifelong anticoagulation with warfarin, exposing patients to bleeding risks and lifestyle limitations [38]. The Ross procedure provides an excellent alternative with superior long-term outcomes and freedom from anticoagulation, though its technical complexity restricts availability to specialized centers with experienced surgeons [28].

For middle-aged patients and those over 60, the decision between mechanical and bioprosthetic valves has become increasingly nuanced. The rapid evolution of transcatheter therapies has fundamentally shifted the treatment paradigm toward bioprosthetic SAVR with planned future valve-in-valve TAVR [28,38]. This strategy allows patients to avoid the burden of lifelong anticoagulation associated with mechanical valves, particularly valuable when the Ross procedure is not feasible, while accepting the likelihood of future reintervention [28]. The trade-off has become increasingly favorable as TAVR technology advances and outcomes improve.

When SAVR is selected as the initial strategy, specific anatomical and technical considerations become crucial for both immediate outcomes and future reintervention feasibility. SAVR optimization requires deliberate planning to facilitate potential valve-in-valve TAVR in the future, while ensuring excellent primary results [11].

The primary advantage of SAVR-first strategy lies in performing surgery when patients are at their lowest surgical risk, with in-hospital mortality of 1.4% among low-risk patients [39]. However, optimization extends beyond immediate outcomes to encompass lifetime management considerations. Key factors include prosthesis selection, sizing to prevent patient-prosthesis mismatch, and anatomical modifications to ensure future transcatheter options remain viable [11].

## 12. Aortic Root Enlargement

Aortic root enlargement represents an important consideration for patients with small annuli who may require future valve-in-valve procedures. Several surgical techniques facilitate implantation of valves larger than the native annulus would normally accommodate, typically achieving enlargement of one to two valve sizes [29]. The Y-incision aortic annular enlargement technique has proven particularly effective, enabling upsizing by three to four valve sizes when necessary [30].

While root enlargement carries similar mortality to isolated AVR in experienced centres, it increases operative complexity with longer cross-clamp/cardiopulmonary bypass time and higher bleeding risk, and it introduces patch-related failure modes (e.g., dehiscence or pseudoaneurysm) that may complicate future reinterventions [40]. The trade-off may be worthwhile from a lifetime management perspective because larger surgical valves reduce the risk of severe patient–prosthesis mismatch during future valve-in-valve TAVR [12]. Balloon valve fracture after aortic root enlargement is considered high risk due to potential root or patch rupture and is not recommended [12].

Current trends show increasing use of root enlargement procedures, rising from 3.9% to 6.3% in recent US data [29]. This trend likely reflects growing awareness of lifetime management principles, as root enlargement during the index procedure may prevent the need for high-risk reoperations or complex transcatheter interventions in the future [11,29].

## 13. Prosthesis Selection for Future Valve-in-Valve

Surgical bioprostheses are categorized as stented, stentless, or sutureless, with stented valves representing the most common choice [11,32]. Understanding specific valve characteristics proves essential for future valve-in-valve planning. The true internal diameter varies significantly based on whether leaflets are porcine or pericardial and whether they are mounted inside or outside the frame [32]. Therefore, knowledge of the implanted valve type is crucial for predicting feasibility of future valve-in-valve intervention [11].

The Inspiris valve offers unique advantages for future interventions, with 19–25 mm sizes featuring an expansion zone that allows implantation of a TAVR valve one size larger [11]. However, this feature theoretically increases coronary obstruction risk if sinus dimensions are inadequate, requiring careful preprocedural assessment [11].

Stentless valves present particular challenges for future valve-in-valve procedures. While they offer excellent haemodynamics with low post-implantation gradients, they fail predominantly through regurgitation and extensive root calcification, making both valve-in-valve TAVR and redo surgery technically challenging [33]. Their use should be carefully considered in the context of lifetime management.

## 14. Preventing Future Coronary Obstruction

Coronary obstruction represents a life-threatening complication significantly more common after valve-in-valve TAVR than native valve TAVR. Key anatomical risk factors include: coronary height < 12 mm from the annular plane, narrow sinotubular junction, and shallow sinuses of Valsalva < 30 mm in diameter [11].

During SAVR, specific techniques can reduce future coronary obstruction risk. When anatomy suggests high risk (particularly with low coronary heights and narrow sinuses), avoiding stentless bioprostheses and stented valves with externally mounted leaflets becomes central, as these valve types create higher leaflet profiles that increase obstruction risk during future valve-in-valve procedures [11].

For patients with particularly challenging anatomy and appropriate life expectancy, some centers suggest considering aortic root replacement using Valsalva grafts with large sinuses relative to valve size in order to facilitate future valve-in-valve TAVR in roots with otherwise unfavorable anatomy, though it increases the complexity of the index procedure [11].

## 15. Optimizing for Future Patient Prosthesis Mismatch

Patient-prosthesis mismatch following valve-in-valve procedures occurs in up to 30% of cases and significantly impacts outcomes [32,34]. Risk factors include small surgical valve size (particularly internal diameter < 20 mm), stenosis as the mechanism of degeneration, and inadequate initial valve sizing [32]. Therefore, facilitating the largest possible bioprosthesis during the initial SAVR, including root enlargement, is recommended (ideally to achieve a minimum 23 mm prosthesis size) [11].

Preoperative CT is not mandated for all patients undergoing surgical bioprosthetic AVR, but it is increasingly used in selected patients (e.g., small annulus, complex root anatomy, or anticipated future valve-in-valve) to improve annular sizing, anticipate patient–prosthesis mismatch, and plan root enlargement. Recent data suggest CT-based sizing may reduce valve undersizing and mismatch and can support virtual valve-in-valve simulation to anticipate coronary obstruction and residual gradients [41,42,43].

## 16. Special Considerations

Concomitant atrial fibrillation may deserve particular attention during SAVR planning. A retrospective review of American Medicare data demonstrated a survival and stroke-freedom benefit with SAVR and concomitant AF treatment (e.g., left atrial appendage obliteration, surgical ablation) compared to TAVR alone [35]. Prospective trials addressing this population should be performed before recommending any changes in practice.

Infective endocarditis after TAVR is uncommon but serious. Contemporary series report an incidence of approximately 0.3–2.0 per 100 person-years, with rates similar to surgical bioprosthetic AVR and persistently high morbidity and mortality [44]. As TAVR expands to younger patients, prevention (including oral health optimization and guideline-directed prophylaxis in high-risk scenarios), early recognition, and multidisciplinary endocarditis team management remain essential.

The evolving landscape of cardiac surgery reflects lifetime management principles. SAVR volumes have shifted toward more complex cases in dedicated centers, with increasing rates of concomitant procedures and root interventions [11]. Development of dedicated valve centers likely promotes optimal outcomes for both the index procedure and future reinterventions.

In summary, optimizing the initial SAVR requires balancing immediate surgical outcomes with long-term reintervention feasibility. Strategic use of root enlargement, careful prosthesis selection, and anatomical modifications to prevent future complications represent key components of successful lifetime management. These considerations should be integrated into preprocedural planning using CT imaging and multidisciplinary team evaluation to ensure optimal outcomes throughout the patient’s lifetime.

## 17. Valve-in-Valve TAVR: Planning and Execution

When the index valve fails, valve-in-valve TAVR has emerged as the preferred reintervention strategy when anatomically feasible. Success rates exceed 85% with 30-day mortality of 3–6% in appropriately selected patients, comparing favorably to surgical explantation, which carries mortality rates of 13–20% [45,46]. However, valve-in-valve procedures introduce unique technical challenges requiring meticulous CT-based planning and execution.

## 18. TAVR-in-SAVR Considerations

Valve-in-valve TAVR within failed surgical bioprostheses represents the most established reintervention pathway. Understanding the specific surgical valve characteristics proves essential, as labeled sizes do not correspond to true internal diameters. Stented valves with leaflets mounted inside the frame provide larger effective orifices than those with externally mounted leaflets, directly impacting feasibility and outcomes [11].

CT assessment for TAVR-in-SAVR must evaluate multiple parameters. First, the true internal diameter of the surgical valve determines maximum TAVR size and predicts residual gradients. Valves with internal diameter < 20 mm carry high risk for severe PPM, occurring in up to 27% of valve-in-valve cases compared to 5% in native valve TAVR. Second, the valve-to-coronary distance requires careful measurement, with <4 mm predicting coronary obstruction. Third, the relationship between surgical valve posts and coronary ostia must be assessed, as unfavorable orientation may preclude safe valve-in-valve despite adequate distances [36,47].

Virtual valve simulation has become indispensable for predicting valve-in-valve outcomes. Using CT data, operators can simulate THV deployment within the surgical valve, assessing predicted gradients, identifying coronary obstruction risk, and determining optimal valve sizing. The VIV application [48] provides valve-specific information, including true internal diameter, optimal THV selection, and deployment recommendations. This tool has significantly improved procedural planning, with studies showing excellent correlation between predicted and actual outcomes [48,49].

Management of acute valve-in-valve procedural failure due to THV malposition or embolization is guided mainly by case series and expert practice. When malposition is recognized early with a repositionable system, partial recapture and redeployment may be possible. For significant migration or embolization, bail-out options include stabilizing the first valve with a second THV (valve-in-valve rescue) and/or snare-assisted repositioning. Hemodynamic collapse or inability to secure the valve mandates urgent surgical retrieval/explant. These scenarios highlight the value of meticulous CT sizing, controlled deployment, and use of clear fluoroscopic depth markers.

Risk stratification for coronary obstruction in TAVR-in-SAVR differs from native valve procedures. As previously mentioned, bioprosthesis-related risk factors include supra-annular valve position, high leaflet profile, and use of stentless or stented valves with externally mounted leaflets. Patient-specific factors include coronary height < 12 mm, sinotubular junction diameter < 30 mm, and shallow sinus of Valsalva. When virtual valve-to-coronary distance measures < 4 mm, coronary protection strategies or BASILICA should be considered [11,50].

## 19. TAVR-in-TAVR Considerations

Redo-TAVR within a failed transcatheter valve presents unique technical challenges distinct from valve-in-valve procedures in surgical bioprostheses. Planning centers on the neoskirt/risk plane concept—the cylindrical zone created when redo deployment pins the index valve leaflets against the aortic wall—because it influences coronary access and obstruction risk [16,51,52]. In contemporary practice, feasibility also depends on valve-to-coronary (VTC) and valve-to-sinotubular junction (VTSTJ) clearance and the achievable implantation depth of the index and redo devices.

The risk plane height is estimated by subtracting the index implantation depth from the transcatheter valve leaflet (or commissural) height. This provides a practical starting point for anticipating whether coronaries and the sinotubular junction will remain accessible after redo implantation [16,51,52,53].

Importantly, implantation depth is one of the few modifiable determinants of future neoskirt height, but each valve platform has a practical depth range constrained by anchoring, hemodynamics, paravalvular leak, and conduction safety. Lifetime planning, therefore, requires balancing a target depth that preserves future coronary access with immediate procedural risks, rather than treating risk plane thresholds in isolation.

Valve design significantly influences risk plane characteristics. Balloon-expandable SAPIEN 3 valves feature leaflets that extend to the commissural tab apex, creating a relatively predictable neoskirt height [49]. In contrast, self-expanding platforms (Evolut, ACURATE neo2, Portico) incorporate leaflets reaching the full commissural post height, generating substantially taller neoskirts. The supra-annular architecture and extended frame length of self-expanding prostheses further amplify this effect, creating additional planning complexity [15,40].

CT planning for TAVR-in-TAVR requires systematic evaluation of multiple factors:

- Coronary access and obstruction risk: Ideally, the projected neoskirt remains below the coronary ostia. When the risk plane lies between coronary height and the STJ, redo feasibility depends on adequate valve-to-coronary (VTC) distance and accessible frame cells; pragmatic thresholds such as VTC > 4 mm have been used in virtual simulation datasets but are platform- and technique-dependent [16,52]. If the risk plane extends above the STJ, valve-to-STJ clearance becomes critical (e.g., VTSTJ > 2 mm in some datasets), and coronary inaccessibility may occur even when coronary heights are not low [16,52].
- Neoskirt Management: Post-TAVR CT studies by Grubb and colleagues [54] demonstrated valve-specific strategies for managing neoskirt height. For index Evolut valves, implanting a SAPIEN 3 at low position (node 4) resulted in only 20% high-risk cases for coronary compromise. Conversely, tall-frame valve combinations predictably produce the highest risk planes. Understanding these interactions guides both initial valve selection and subsequent reintervention planning [54,55].
- Patient Prosthesis Mismatch Risk: The “Russian doll” effect of multiple valves significantly increases PPM risk, particularly in small annuli. Fukui et al. found that using two SAPIEN 3 valves resulted in 21% incidence of at least moderate PPM compared to 1% with mixed platforms. CT-based calculation of predicted indexed EOA becomes crucial, using established formulas accounting for both valves’ contributions to flow restriction [16,56].

## 20. Technical Optimization Strategies

Several technical considerations optimize valve-in-valve outcomes. In a retrospective study, Ochiai and colleagues demonstrated that high implants (1–3 mm of THV implantation depth) increased sinus sequestration risk but reduced conduction abnormalities, while low implants (3–5 mm) improved coronary access at the cost of increased pacemaker rates [57]. This highlights the need for individualized depth selection based on patient-specific anatomy and priorities.

Commissural alignment during the index procedure proves crucial but challenging to maintain during redo-TAVR. Ex vivo studies demonstrate that strut misalignment reduces accessible cell dimensions by up to 22%, with misaligned self-expanding combinations producing the smallest accessible areas [58]. While perfect alignment cannot be guaranteed, awareness of this limitation influences initial valve selection in patients likely to require reintervention [22,58].

The phenomenon of index valve re-expansion following second valve deployment requires consideration. Bench studies show that balloon-expandable valves implanted within self-expanding platforms can increase the index valve waist diameter by up to 2.5 mm, potentially transforming a feasible procedure into one with high obstruction risk. Pre-procedural modeling should account for this expansion when borderline anatomy exists [49,59].

## 21. Leaflet Modification Techniques

As previously described in this review, when standard valve-in-valve approaches are deemed high-risk, leaflet modification may enable treatment. The BASILICA technique, while well-established for surgical valves, faces challenges in THVs due to leaflet redundancy and design differences [41,42]. Success depends critically on index valve commissural alignment and depth, with misaligned commissures potentially obstructing coronaries despite successful laceration [41,60,61,62].

Novel dedicated devices are being explored to simplify leaflet modification, but the current evidence base remains early. The ShortCut device has been evaluated in first-in-human and early feasibility experiences (approximately 60 patients) with high acute success and low early adverse events; however, these reports reflect selected centers and short follow-up and should be interpreted as preliminary [35,63]. Appropriate patient selection remains crucial, and CT simulation is essential to confirm that leaflet splitting will create an adequate flow channel for the coronary ostium [54,63]. The UNICORN technique offers an alternative approach using a radiofrequency needle to traverse and lacerate the leaflet, and has been adapted for different valve platforms [34]. The LLAMACORN modification (Leaflet Laceration with bAlloon Mediated Annihilation to prevent Coronary Obstruction with Radiofrequency Needle) combines radiofrequency traversal with sequential balloon dilation to facilitate safe deployment of self-expanding valves, but published experience remains limited to small observational series [64,65]. Collectively, these emerging strategies may be useful in selected anatomies where traditional BASILICA is challenging; broader generalizability and durability of outcomes require larger registries and longer-term follow-up.

## 22. SAVR After Failed TAVR

When valve-in-valve TAVR is not feasible, surgical options must be considered. TAVR explantation represents a complex, high-risk procedure with 30-day mortality of 13.1% in the EXPLANT-TAVR registry. For younger patients or those with anatomy precluding safe valve-in-valve procedures, planning for eventual surgical explantation influences initial valve selection. Balloon-expandable valves generally facilitate easier extraction due to their shorter frames and predictable anatomical interaction [11]. Self-expanding TAVRs pose particular challenges for explantation due to frame endothelialization within the aortic wall, often necessitating root or ascending aortic replacement [66,67,68].

## 23. Future Directions

Advanced Valve Design

Contemporary valve development specifically addresses lifetime management challenges through targeted innovations in both balloon-expandable and self-expanding platforms. Recent advancements in balloon-expandable technology, exemplified by the SAPIEN X4 (Edwards Lifesciences), incorporate multiple design enhancements: proprietary anti-calcification technology aimed at extending valve longevity, enlarged frame cell geometry facilitating improved coronary access for future interventions, reduced overall frame height minimizing coronary obstruction risk, and adjustable sizing algorithms that maintain optimal hemodynamics across diverse anatomies [69,70]. The ongoing ALLIANCE trial (NCT04415047) will provide critical safety and efficacy data to validate these design improvements in clinical practice [69].

Self-expanding platforms demonstrate parallel evolution in addressing lifetime management considerations. The current Evolut FX Plus system (Medtronic) has established improvements for precise positioning through enhanced radiopaque commissural alignment markers and addresses coronary access challenges with its large cell design, building upon iterative refinements in catheter deliverability [71,72]. These design refinements reflect growing recognition that initial valve selection must account for potential future coronary interventions and redo procedures, particularly as manufacturers optimize their platforms to facilitate both commissural alignment and maintained coronary access throughout the patient’s lifetime.

Novel materials science approaches promise to address fundamental durability limitations. The Foldax TRIA valve employs proprietary synthetic polymer technology designed to resist calcification and thrombus formation, potentially offering lifelong durability without animal-derived tissue [69]. Similarly, BCI’s polyphenol-based coating technology demonstrates up to 60% reduction in thrombus formation and 99% reduction in protein adhesion compared to untreated materials, suggesting significant potential for extending valve lifespan [73,74,75]. The DurAVR system [51] utilizes ADAPT-treated bovine pericardium with single-piece leaflet construction to minimize stress concentrations and reduce calcification risk. These material innovations may prove particularly valuable for younger patients requiring multiple interventions over their lifetime.

## 24. Computational Modelling and Artificial Intelligence

The integration of artificial intelligence (AI), machine learning, and patient-specific computational modelling is emerging as a useful adjunct for procedural planning and risk stratification. Most published models have been developed and tested retrospectively using single-centre or multicenter imaging datasets and have focused on predicting discrete procedural endpoints such as paravalvular regurgitation, new conduction abnormalities/permanent pacing, vascular complications, or mortality. Reported discrimination is encouraging (often AUC in the ~0.75–0.90 range), and automated segmentation/measurement tools can reduce inter-observer variability; however, external validation, calibration across valve platforms, and prospective impact studies demonstrating improved outcomes remain limited [76].

Patient-specific finite element and computational models can simulate valve deformation patterns, calcium displacement trajectories, and frame–LVOT contact pressures to derive metrics such as the contact pressure index and predict conduction disturbance risk [16,76]. These analyses are primarily modelling-based, and their performance depends on assumptions regarding material properties, implantation depth, and valve platform; therefore, translation to routine practice will require streamlined workflows and prospective validation.

Image-fusion techniques that register pre-procedural CT datasets to real-time fluoroscopic guidance can assist with commissural alignment, optimal implantation depth targeting, and real-time appreciation of coronary ostial relationships [77]. Available evidence is largely from early feasibility studies and selected cohorts; potential benefits include reduced procedure time, contrast volume, and radiation exposure, but robust multicenter outcome data are still evolving [78].

The convergence of AI, computational modelling, and advanced imaging represents a paradigm shift toward precision medicine in structural heart interventions. Future iterations may incorporate real-time hemodynamic modelling, automated complication detection, and predictive algorithms for long-term outcomes. Integration with electronic health records could enable continuous learning systems that refine predictive accuracy based on accumulating clinical experience. As these technologies mature, they promise to optimize both immediate procedural success and lifetime management strategies, ensuring each patient receives truly personalized care based on their unique anatomical and clinical characteristics.

## 25. Personalized Lifetime Risk Assessment

Development of integrated risk calculators incorporating patient-specific factors, anatomical measurements, and procedural variables would significantly enhance decision-making [32]. Such tools could estimate reintervention probability across different initial strategies, predict feasibility of various reintervention options, and guide timing of interventions based on valve degeneration patterns. The STS risk calculator’s recent incorporation of TAVR explantation represents an important step toward comprehensive lifetime risk assessment (STS Database) [11].

## 26. Standardization of Care Pathways

Standardized approaches to lifetime planning should become routine. This includes mandatory pre-procedural CT analysis with specific attention to future reintervention feasibility, multidisciplinary team evaluation incorporating lifetime risk assessment, and systematic documentation of implantation parameters, including depth, orientation, and final valve dimensions. Creation of centralized registries tracking long-term outcomes and reintervention patterns would further inform evidence-based protocols [15].

The establishment of specialized valve centers with expertise in complex reinterventions, including TAVR explantation and advanced leaflet modification techniques, will become increasingly important. These centers can serve as referral destinations for challenging cases while advancing technique development and training.

## 27. Conclusions

The lifetime management of TAVR patients requires a fundamental shift from focusing solely on immediate procedural success to considering long-term implications of initial treatment decisions. Comprehensive CT planning enables optimization of the index procedure while preserving future options. The decision between TAVR and SAVR as initial therapy must incorporate patient preferences, anatomical considerations, and lifetime risk assessment rather than age alone.

When TAVR is selected, meticulous attention to valve selection, sizing, and positioning can minimize complications while facilitating future interventions. Understanding concepts like the risk plane and neoskirt height proves essential for predicting redo-TAVR feasibility. As valve technology advances and our understanding of long-term outcomes improves, personalized strategies will continue to evolve.

Successful lifetime management demands multidisciplinary collaboration, combining clinical expertise with advanced imaging and computational modeling. The goal remains clear: optimizing outcomes not just for the index procedure, but throughout each patient’s lifetime journey with aortic valve disease. Future research should focus on long-term durability data, refinement of reintervention techniques, and development of integrated risk assessment tools to guide individualized care.

## Figures and Tables

**Table 1:** Summary of high-impact predictors and adjunct planning metrics relevant to index TAVR optimization and redo feasibility.

Planning Domain	High-Impact Predictors (Primary Drivers)	Adjunctive Modifiers and Planning Metrics
Patient-prosthesis mismatch (index and redo)	Small annulus relative to body surface area (low predicted indexed EOA); small THV size; compounding restriction from nested valves (“Russian doll” effect)	Supra-annular versus intra-annular leaflet position; implantation depth; predicted EOA models; CT image quality, artefact, and heavy calcification
Paravalvular regurgitation	High calcium burden with adverse distribution (LVOT, annular, bulky nodules, marked asymmetry)	Annular eccentricity and ellipticity; cover index and oversizing strategy; sealing skirt design; need for balloon pre- or post-dilatation; deployment angle
Conduction disturbance and permanent pacing	Short membranous septum with deep implantation relative to membranous septum length; baseline conduction disease; LVOT calcium at the NCC-RCC interface	Valve platform and radial force; cusp-overlap or high-implant technique; degree of oversizing; post-dilatation; anticipated pacing strategy
Coronary obstruction and sinus sequestration	Low coronary height with small sinus dimensions or narrow sinotubular junction; long native leaflet length; small virtual valve-to-coronary distance	Valve-to-sinotubular junction clearance; commissural alignment; implantation height; leaflet calcium distribution; choice of coronary protection or leaflet modification strategy
Annular rupture	Subannular and LVOT calcification (particularly nodular); aggressive oversizing with balloon expansion (balloon-expandable platforms)	Balloon type and sizing; predilatation strategy; self-expanding versus balloon-expandable platform selection; avoidance of excessive post-dilatation
Redo-TAVR feasibility and coronary re-access	Neoskirt or risk plane above coronary ostia or sinotubular junction with limited valve-to-coronary or valve-to-sinotubular junction clearance; tall commissural posts	Achievable implantation depth of the index valve; commissural alignment strategy; valve-pairing effects including index valve re-expansion; CT simulation and catheter access planning

## Data Availability

No new data were created or analyzed in this study.
